# Evaluation of the InTray and Compact Dry culture systems for the diagnosis of urinary tract infections in patients presenting to primary health clinics in Harare, Zimbabwe

**DOI:** 10.1007/s10096-021-04312-4

**Published:** 2021-07-22

**Authors:** Ioana D. Olaru, Wael Elamin, Mutsawashe Chisenga, Nada Malou, Jeremie Piton, Shunmay Yeung, Rashida A. Ferrand, Heidi Hopkins, Prosper Chonzi, Kudzai P. E. Masunda, Portia Manangazira, Cecilia Ferreyra, Katharina Kranzer

**Affiliations:** 1 Clinical Research Department, London School of Hygiene and Tropical Medicine, Keppel Street, Bloomsbury, London, WC1E 7HT UK; 2 Biomedical Research and Training Institute, Harare, Zimbabwe; 3 G42 Healthcare, Abu Dhabi, United Arab Emirates; 4 Queen Mary University London, London, UK; 5 Elrazi University, Khartoum, Sudan; 6 FIND (Foundation for Innovative New Diagnostics), Geneva, Switzerland; 7 Department of Paediatric Infectious Disease, St. Mary’s Imperial College Hospital, London, UK; 8 City of Harare, Health Department, Harare, Zimbabwe; 9 Ministry of Health and Child Care, Harare, Zimbabwe; 10 Division of Infectious and Tropical Medicine, Medical Centre of the University of Munich, Munich, Germany

**Keywords:** AMR, Antibiotic resistance, *Enterobacterales*, ESBL

## Abstract

Antimicrobial resistance surveillance data is lacking from many resource-limited settings mainly due to limited laboratory testing. Novel culture systems may address some of the limitations of conventional culture media and expand the availability of microbiology services. The aims of this study were to evaluate the performance of InTray COLOREX Screen/ESBL and Compact Dry for the detection of uropathogens and of extended-spectrum beta-lactamase (ESBL)-producing organisms from urine samples. Urines samples were collected from patients presenting with symptoms of urinary tract infection to primary care clinics in Harare. Performance of the InTray COLOREX Screen, ESBL and Compact Dry chromogenic media were compared to the reference of culture using Brilliance UTI agar and conventional antimicrobial susceptibility testing. A total of 414 samples were included in the analysis. Of the included samples, 98 were positive on Brilliance UTI agar and 83 grew *Enterobacterales*. The sensitivities and specificities for *Enterobacterales* were 89.2% (95% CI 80.4–94.9) and 98.2% (95% CI 96.1–99.3) for InTray Screen and 95.2% (95% CI 88.1–98.7) and 99.7% (95% CI 98.3–100) for Compact Dry. Extended-spectrum beta-lactamases were present in 22 isolates from the Brilliance UTI agar. The sensitivity of the InTray COLOREX ESBL culture plates for the detection of ESBL-producing organisms was 95.5% (95% CI 77.2–99.9) and specificity was 99.5% (95% CI 98.2–99.9%). Our findings show good performance of the novel culture systems for the detection of uropathogens and ESBL-producing organisms. Both systems have several advantages over conventional media and have the potential to expand and decentralize laboratory testing.

**Supplementary Information:**

The online version contains supplementary material available at 10.1007/s10096-021-04312-4.

## Introduction

Antimicrobial resistance (AMR) represents a major threat to human health by impeding effective treatment of serious infections and leading to increased morbidity, mortality and healthcare costs [1]. Acknowledging the implications of rising AMR, in 2015, the World Health Assembly adopted the Global Action Plan on AMR which outlined specific actions to address the increase in AMR including strengthening AMR surveillance and global data sharing [2]. In response to the Global Action Plan, the World Health Organization launched the Global AMR Surveillance System (GLASS).

The 2020 GLASS report emphasizes the persisting geographic gaps in AMR surveillance with health facilities and laboratories from Europe and the Americas contributing the majority of data. Data from low- and middle-income countries (LMIC) in Africa and Southeast Asia are scarce due to limited laboratory capacity and availability of microbiological diagnosis [3]. The main challenges restricting bacterial identification and detection of resistance are insufficient laboratory scientists and technicians, stock-outs of laboratory consumables, short shelf life of some key reagents, difficulties in supply chains management and prohibitive costs [4, 5]. In most LMICs, both availability and cost of diagnostic tests limit access for a large majority of patients who have most infectious conditions treated empirically and with the use of case definitions rather than laboratory confirmation. Blood cultures for example are usually restricted to tertiary referral hospitals and are not available at lower tier hospitals (i.e. district hospitals) and peripheral healthcare facilities [6].

Even at the levels where these tests are available, the turnaround time is such that antimicrobials are prescribed and consumed before the results are available to influence the choice. Limited availability of diagnostics results in a lack of AMR surveillance data and non-adaptation of empirical treatment to local pathogens and resistance profiles. Available data are usually biased towards more complex cases treated at referral centres or in private healthcare settings, and are not representative of the overall burden of resistance, challenging the development of locally adapted treatment recommendations.

Two novel ready-to-use culture systems, InTray and Compact Dry, may facilitate processing of urine samples in low-resource settings particularly in regional and district healthcare facilities where fully equipped microbiology laboratories may not be available. These systems have particular advantages that may allow for their use in lower tier facilities, thus expanding access to microbiology services. Urinary tract infections (UTIs) are common Gram-negative infections in outpatient settings. Organisms causing UTIs may provide valuable information on community-level Gram-negative resistance. Urine samples are non-invasive and considered priority specimens for AMR surveillance by the WHO-GLASS [3].

The aims of this study were to evaluate the performance of InTray COLOREX Screen/ESBL and Compact Dry for the detection of uropathogens and of extended-spectrum beta-lactamase (ESBL)-producing organisms in urine samples from patients presenting with UTI symptoms to primary care clinics in Harare.

## Methods

Study participants were recruited into the antimicrobial resistance in Gram-negative bacteria from Urinary Specimens (ARGUS) study which is an observational cross-sectional study enrolling adult patients who present with suspected UTIs to one of nine participating primary health clinics in Harare. The procedures and eligibility for the ARGUS study have been described in detail elsewhere [7]. Briefly, after obtaining informed consent, demographic and medical history was collected using a questionnaire and entered directly in an electronic form using the Open Data Kit (ODK, www.opendatakit.org). A midstream urine sample was collected from each of the study participants. If the transportation time to the laboratory was anticipated to exceed 4 h, the urine sample was placed in a thermally insulated bag containing ice packs.

### Culture media

This study used two novel culturing techniques—(1) InTrays COLOREX Screen and InTray COLOREX ESBL (Biomed Diagnostic, White City, OR, USA) and (2) Compact Dry EC (Nissui Pharmaceutical Co. Ltd., Tokyo, Japan)—and compared them to culture on Brilliance UTI agar (Oxoid, UK) which is considered the reference standard [8].

InTrays are ready-to-use commercially available small-sized agar plates (5-cm diameter for the agar) with chromogenic substrates to differentiate between multiple bacterial species based on colony colour. InTray COLOREX ESBL plates contain, in addition, antimicrobial compounds for selective identification of extended-spectrum beta-lactamase (ESBL)-producing organisms. InTrays have to be stored at 2–8 °C and have a shelf life of 6 (InTray COLOREX ESBL) to 12 months (InTray COLOREX Screen) [9].

Compact Dry EC is a dehydrated ready-to-use chromogenic medium designed for quantifying *Escherichia coli* and coliforms from food products. The media is hydrated by sample inoculation and capillary action allows for diffusion of the sample across the plate. The media contains two chromogenic enzyme substrates, Magenta-Gal and X-Gluc, which enable the differentiation between *E. coli* (blue) and other coliforms (pink). The plastic casing has a grid with large and small squares for ease of colony counting. For optimal colony counting, the manufacturer recommends diluting samples to concentrations of 100 colony forming units (CFU)/mL. The media is heat, light and moisture-sensitive and is supplied in small opaque pouches containing desiccant. The plates are stored at room temperature up to 30 °C and have a shelf life of 18 months post-manufacture [10].

Colony appearance and characteristics of the different media are described in Table 1 and additional Figures S1 and S2.

**Table 1:** Colony appearance and product characteristics for the three culture systems used in the study [8–10]

	Brilliance UTI agar	InTray COLOREX	Compact Dry EC
*E. coli*	Pink	Pink	Blue
KESC*	Dark blue	Blue/turquoise	Red-violet (pink)
*Proteus group*	Brown halo	Brown halo	Yellow–brown
*Pseudomonas spp.*	Brown/green	Cream, translucent	-
*Enterococcus spp.*	Turquoise	Blue/turquoise	Inhibited
*S. aureus*	White/cream	Golden	Inhibited
*S. saprophyticus*	Pink, small	Pink, small	Inhibited
Other streptococci/staphylococci	Non-pigmented/white	White	Inhibited
Product and use characteristics			
Storage temperature	Dehydrated: RT Prepared: 2–8 °C	2–8 °C	RT (< 30 °C)
Shelf life	Dehydrated: ~ 2 years Prepared: 2 weeks	6–12 months	18 months
Size/format (mm)	Dehydrated: container Prepared: 85 × 85 × 14	103 × 75 × 8 Agar: 5 cm	Culture media: 5.5 cm
Number of samples	Multiple	Single	Single
Preparation required	Yes (dehydrated)	No	No
Number of samples inoculated	1–6/ plate	1	1
Sample and volume inoculated	Neat urine, 1 µL	Neat urine, using a swab	Diluted urine 10^3^ and 10^6^; 1 mL
Location of sample inoculation	Laboratory	Point of care (clinic)	Laboratory

*KESC Klebsiella, Enterobacter, Serratia, Citrobacter*; *RT* room temperature*.* Dehydrated media, supplied in powder form requiring preparation; prepared, pre-poured plates as supplied by the manufacturer

### Sample processing

Following collection, the urine samples were inoculated by trained research assistants at the point of care onto the InTray COLOREX Screen and ESBL culture plates using a cotton swab (one tray per sample). For the Compact Dry and Brilliance UTI agar, urine samples were processed at the Biomedical Research and Training Institute research laboratory. The inoculated culture plates were then transported to the laboratory where they were incubated at 37 °C for 24 h. The InTrays were read after 24 h by a trained microbiologist. Information on the number of CFU, morphology, colour and growth was recorded using a standardized form. Because InTrays require refrigeration, uninoculated plates had to be transported at the end of each working day to the laboratory and dispatched again to the clinics on the following day.

For Compact Dry, it was assumed that positive urine cultures would have a bacterial concentration of at least 10^3^ CFU/mL [11]. To perform colony counts, serial dilutions of 1:10 were performed on the day of sample collection. The urine samples were diluted up to 10^6^ in sterile phosphate-buffered saline and 1 mL each of the 10^3^ and 10^6^ dilutions was inoculated on the Compact Dry and incubated for 24 h at 37 °C. The optimal dilutions were established during a pilot phase and previous use of the culture system in our laboratory (unpublished). The plates were read by a trained microbiologist and the number of CFU and colony appearance was recorded. If colonies could not be counted, growth was categorized semi-quantitatively into semi-confluent (some individual colonies still visible) and confluent growth (individual colonies not visible with a change in substrate colour).

Brilliance UTI agar was inoculated at the laboratory using 1-µL sterile loops and incubated at 37 °C for 24 h. Growth was reported semi-quantitatively into three categories: 10^3^–10^4^ CFU/mL, 10^4^–10^5^ CFU/mL and > 10^5^ CFU/mL. All cultures showing growth of > 10^3^ CFU/mL with the predominance of uropathogens were considered positive.

Antimicrobial susceptibility testing (AST) was done by disc diffusion and interpreted according to the EUCAST standards [12]. ESBL testing of *Enterobacterales* was performed according to the EUCAST recommendations [13]. Briefly, isolates were screened for the presence of ESBLs using cefpodoxime. Isolates positive on the screening test underwent confirmation by synergy testing with amoxicillin/clavulanic acid and ceftazidime. Quality control for bacterial identification and AST was performed using ATCC reference isolates.

### Experience in using the tests

Research assistants and laboratory staff involved in sample processing were asked about the perceived advantages and shortcomings of the test systems. The number of times the InTray plates were removed from the fridge and dispatched to the clinics was recorded to determine if multiple exposures to high temperatures affect the test performance.

### Statistical analysis

Descriptive analyses were performed in STATA v.15 (StataCorp, TX, USA). To evaluate test performance, sensitivity and specificity with 95% confidence intervals (95% CI) were calculated. The correlation between CFUs on Compact Dry and growth on Brilliance UTI agar categorized semi-quantitatively was evaluated using Spearman’s correlation test.

The study was granted ethics approval by the London School of Hygiene and Tropical Medicine (ref. 16,424) and by the Medical Research Council of Zimbabwe (MRCZ/A/2406).

## Results

A total of 431 urine samples were tested using the three culture systems. Of those, 17 (3.9%) were contaminated on Brilliance UTI agar and were excluded leaving 414 samples for the final analysis. The median age of study participants was 36 years (IQR 26–46), 263 (63.5%) were female and 169 (42.7%) were HIV positive. Participant characteristics are shown in additional Table S1.

### InTray COLOREX Screen for uropathogen detection

Using the Brilliance UTI agar, 98 urine cultures were positive. Uropathogens identified were 72 *E. coli* (73.5%), 11 other *Enterobacterales* (11.2%), 13 *Enterococcus spp.* (13.3%) and two *Staphylococcus aureus* (2.0%). Of the 72 cultures growing *E. coli* on the Brilliance UTI agar, 64 showed growth of pink colonies (*E. coli*) on the InTray COLOREX Screen culture plates while eight were positive on Brilliance only and six on InTrays only. For other coliforms, ten cultures were positive on both Brilliance and InTrays and an additional sample was positive on the Brilliance UTI agar only (Table 2).

**Table 2:** Comparison between culture results for InTray Screen and Brilliance UTI agar for *Enterobacterales*

		Brilliance UTI agar				Total
		Negative for coliforms^#^	10^3^–10^4^ CFU/mL	10^4^–10^5^ CFU/mL	> 10^5^ CFU/mL	
InTray COLOREX Screen	Negative (or 1–4 colonies)^*^	325	4	3	2	334
	Positive					
	5–49 colonies	4	5	3	0	12
	50–100 colonies	0	1	0	1	2
	Confluent growth	2	0	8	56	66
	Total	331	10	14	59	414

^*^Negative cultures, one colony on InTray ESBL but not on Screen (*n* = 2); mixed growth of organisms with colonies on InTray but < 5 colonies (*n* = 3); ^#^3/6 discordant cultures with a positive InTray also had growth with low colony count on Compact Dry. Gram-positive organisms (*Enterococcus* spp., *n* = 13 and *S. aureus*, *n* = 2) were not included in the table as their growth is inhibited on the Compact Dry media

Of the 13 samples that were positive on the Brilliance UTI agar for enterococci, nine were also positive for enterococci on the InTray COLOREX Screen cultures. The four discordant samples that were positive for enterococci on the Brilliance UTI agar had growth of enterococci on the InTrays but enterococci were not the dominant organism. Conversely, 15 cultures had predominant growth of enterococci on the InTray but were negative on the Brilliance UTI agar. The two cultures with growth of *S. aureus* were positive on both culture media.

Using the Brilliance UTI agar as the reference standard and a threshold for positivity of 10^3^ CFU/mL, the sensitivity of InTray COLOREX Screen for detecting *Enterobacterales* was 89.2% (74/83; 95% CI 80.4–94.9) and the specificity was 98.2% (325/331; 95% CI 96.1–99.3). Positive and negative predictive values were 92.5% (74/80; 95% CI 84.4–97.2) and 97.3% (325/334; 95% CI 94.9–98.8), respectively. Test performance according to different thresholds for culture positivity is shown in Table 3.

**Table 3:** Performance for Compact Dry EC and InTray Screen in the detection of *Enterobacterales* compared with Brilliance UTI agar and using different thresholds for culture positivity on Brilliance agar

	Cut-off on Brilliance agar	Sensitivity (95% CI)	Specificity (95% CI)	PPV (95% CI)	NPV (95% CI)
Compact Dry at 1:10^3^ dilution	10^3^	95.2 (88.1–98.7)	99.7 (98.3–100)	98.8 (93.2–99.9)	98.8 (97.0–99.7)
	10^4^	98.6 (92.6–100)	97.7 (95.4–99.0)	90.0 (81.2–95.6)	99.7 (98.3–99.9)
	10^5^	100 (93.9–100)	94.1 (91.1–96.3)	73.8 (62.7–83.0)	100 (98.9–100)
InTray COLOREX Screen	10^3^	89.2 (80.4–94.9)	98.2 (96.1–99.3)	92.5 (84.4–97.2)	97.3 (94.9–98.8)
	10^4^	93.2 (84.7–97.7)	96.5 (93.9–98.2)	85.0 (75.2–92.0)	98.5 (96.5–99.5)
	10^5^	96.6 (88.3–100)	93.5 (90.4–95.8)	71.3 (60.0–80.8)	99.4 (97.9–99.9)

*NPV* negative predictive value; *PPV* positive predictive value

### Compact Dry EC for uropathogen detection

**Fig. 1:**
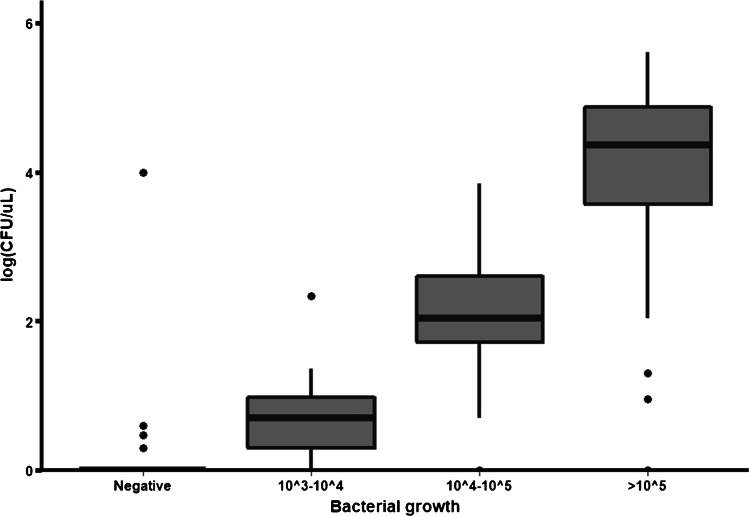
Comparison of semi-quantitative bacterial growth on Brilliance UTI agar and colony counts using Compact Dry

Of the 83 samples which had growth of *Enterobacterales* on Brilliance UTI agar, 79 had growth on the Compact Dry EC of *Enterobacterales* at the 1:10^3^ dilution and 58 at the 1:10^6^ dilution. Four samples which were positive for *E. coli* on Brilliance were negative on Compact Dry and one sample with *Proteus spp.* was positive on Compact Dry only (Table 4). The sensitivity and specificity for uropathogen detection were 95.2% (79/83; 95% CI 88.1–98.7) and 99.7% (330/331; 95% CI 98.3–100), respectively, using a threshold for urine culture positivity of 10^3^ CFU/mL on the Brilliance UTI agar (Table 3). Positive and negative predictive values were 98.8% (79/80; 95% CI 93.2–99.9) and 98.8% (330/334; 95% CI 97.0–99.7). Using the 1:10^3^ dilution, 55/80 (69%) of positive samples on Compact Dry had semi-confluent or confluent growth and therefore colony counts could not be performed. While the 1:10^3^ dilution had a better sensitivity, the higher dilution allowed for colony counts. There was a strong correlation between CFUs determined using the Compact Dry and the semi-quantitative assessment on Brilliance UTI agar (Spearman’s rho 0.924, *p* < 0.001; Fig. 1).

**Table 4:** Comparison between culture results for Compact Dry and Brilliance UTI agar for *Enterobacterales*

		Brilliance UTI agar				Total
		Negative for coliforms	10^3^–10^4^ CFU/mL	10^4^–10^5^ CFU/mL	> 10^5^ CFU/mL	
Compact Dry EC at 1:10^3^ dilution	Negative (or 1–4 colonies)	330	3	1	0	334
	5–49 colonies	0	6	3	2	11
	50–250 colonies	0	1	4	2	7
	Semi-confluent growth or > 250 colonies	0	0	6	18	24
	Confluent growth	1*	0	0	37	38
Compact Dry EC at 1:10^6^ dilution	Negative	331	8	11	6	356
	1–49 colonies^#^	0	2	3	32	37
	50–250 colonies	0	0	0	18	18
	Semi-confluent growth or > 250 colonies	0	0	0	2	2
	Confluent growth	0	0	0	1	1
	Total	331	10	14	59	414

Cultures which showed contamination on Brilliance UTI agar were excluded; *this culture had mixed growth and one of the organisms was Proteus which had confluent growth on the Compact Dry due to swarming; ^#^for the 1:10^6^ dilution, this category included growth of 1–49 colonies

### InTray COLOREX ESBL for the diagnosis of cephalosporin-resistant organisms

InTray COLOREX ESBL culture results were available for 413 samples. Synergy testing for the presence of ESBLs was positive in 22/24 isolates tested (*E. coli*, *n* = 20; other coliforms, *n* = 2). Of the 22 ESBL-positive organisms using conventional methods, 21 were positive on InTray COLOREX ESBL. Two samples were positive on InTray COLOREX ESBL only: negative on Brilliance UTI agar (*n* = 1); negative screening test for ESBL using cefpodoxime (*n* = 1). One sample which was ESBL positive using conventional methods and negative on InTray had growth of a single colony on InTray. The sensitivity of the InTray COLOREX ESBL culture plates for the detection of ESBL-producing organisms was 95.5% (21/22; 95% CI 77.2–99.9) and specificity was 99.5% (400/402; 95% CI 98.2–99.9%).

### Reported experience of staff using the tests

Research assistants who inoculated the urine samples onto InTrays at point of care (*n* = 9) reported that overall the diagnostic device was easy to use, and they reported no challenges inoculating the agar in the field. The procedure was simple and was not time-consuming. The main challenge was the requirement for the InTrays to be refrigerated and thus InTrays not used on a particular day had to be transported daily from the laboratory to the clinics. In order to decrease the number of times the InTrays were transported between the laboratory and clinics, a limited number of InTrays was issued to each clinic every day. This meant that research assistants sometimes ran out of InTrays if the number of participants enrolled outnumbered the number of InTrays sent to the clinic. The median number of times InTrays were sent to the clinics was 2 (IQR 1–4). The number of times an InTray was transported to a clinic did not affect sensitivity of the culture system.

Laboratory staff (*n* = 2) reported on the processing, reading and interpretation of the test results. Both InTray and Compact Dry plates are relatively small in size minimizing incubator space requirements. For both systems, cultures were very easy to read and interpret. A major advantage compared to the UTI Brilliance agar was that InTrays and Compact Dry were ready to use and did not require media preparation and autoclaving. In LMICs, electricity supplies can be unreliable and generators cannot always provide a sufficient power supply for high-energy processes such as autoclaving. In the study setting, media preparation and autoclaving could not be done during power-cuts. However, Compact Dry was more difficult to inoculate because they required sequential dilutions which were performed in the laboratory.

## Discussion

Both novel culture systems performed well in detecting uropathogens compared to Brilliance UTI chromogenic media, the reference standard. The diagnostic systems were easy to use and study staff required minimal training for inoculation and reading. Furthermore, InTray COLOREX ESBL plates showed a high sensitivity and specificity for the detection of ESBL *Enterobacterales* making them an attractive tool for AMR surveillance in LMICs. Because the system selectively identifies ESBL-producing organisms from primary specimens, it can reduce time to results when used for diagnosis, which in turn may reduce time to appropriate therapy.

Conventional culture media have to be prepared frequently and require refrigeration which may be challenging in LMICs due to unreliable electricity supplies and equipment maintenance. Preparation of dehydrated media requires autoclaving and quality control of every prepared batch. InTray and Compact Dry are pre-prepared culture plates which are smaller than conventional plates and have an extended shelf life bypassing some of the shortcomings associated with conventional cultures. Both systems are based on chromogenic identification. Although chromogenic media for bacterial identification are not novel and have been in use for more than two decades [14], media prepared in-house from dehydrated powder usually expire within 2 weeks [8]. InTray COLOREX Screen and ESBL are stable for 6–12 months and Compact Dry EC for 18 months post-manufacture. Furthermore, Compact Dry media does not require refrigeration and can be stored at temperatures up to 30° which is an added advantage when used in LMICs.

The Compact Dry system has been designed for determining bacterial contamination of food [15]. One previous study from Japan reported good sensitivity of the Compact Dry when used to investigate UTIs in humans, but the sample size was small (*n* = 25) [16]. Because the Compact Dry is a highly sensitive culture system and positive urine cultures usually have high bacterial loads, the urine samples were diluted prior to inoculation. For the purpose of this study, serial dilutions were performed. However, the procedures could be simplified by performing a single dilution at a higher factor (for example by diluting 1-µL sample into a 1 mL of sterile solution). A volume of 1 mL is required in order to rehydrate the dry culture media. Although of limited clinical significance, Compact Dry allows for colony counts which can be used to estimate bacterial load in samples. In this study, colony counts using the Compact Dry correlated well with the semi-quantitative results on Brilliance UTI agar.

Gram-positive pathogens such as *Enterococcus* spp. and *S. saprophyticus* account for 15% of uncomplicated UTIs [17]. While InTrays can detect these pathogens, Compact Dry ECs can only be used for the detection of *Enterobacterales* and do not support growth of Gram-positive organisms. Enterococci were detected more frequently using InTrays than on conventional media. This may suggest an under-diagnosis of enterococcal infections using conventional media although growth of enterococci from midstream urine samples may not necessarily reflect the presence of enterococci in the urinary bladder [18] and therefore results should be interpreted with caution considering the patient’s medical history.

Cultures that were positive on InTray only may be explained by inoculation of a larger sample volume using swabs compared to 1-µL inoculation on Brilliance UTI agar. Furthermore, immediate inoculation of the sample may have prevented loss of bacterial viability during transport. False-negative Compact Dry results were only observed for urine samples with lower bacterial load classified as 10^3^–10^4^ CFUs on the Brilliance UTI agar. These urine samples may have shown growth on Compact Dry if the urine had been less diluted (< 10^3^). One sample showed confluent growth for *Proteus* spp. on the Compact Dry but was negative on Brilliance UTI agar. This was due to the inhibition of swarming on the Brilliance agar but not on Compact Dry.

This is the first study evaluating the performance of the InTray and Compact Dry culture systems for the diagnosis of UTI. We acknowledge that the study is limited by its relatively small sample size and low proportion of positive urine cultures.

Our findings show good performance of the novel culture systems for the detection of uropathogens and ESBL-producing organisms. These systems may simplify laboratory workflow, reduce technician processing time and facilitate procurement and stock management. Both systems have potential to expand and decentralize laboratory testing. Use of the systems in sentinel clinical sites may enhance understanding of pathogens and AMR burden in LMICs. Further research is needed to demonstrate cost-effectiveness and feasibility of wider implementation of these systems for AMR surveillance, and potentially impact on patient outcomes in LMICs.

## Supplementary Information

Below is the link to the electronic supplementary material.

Supplementary file1 (DOCX 452 KB)

Supplementary file2 (DOCX 19 KB)

Supplementary file3 (XLS 150 KB)

## Data Availability

The datasets generated and/or analysed during the current study are available in the Data Compass (LSHTM) repository, https://datacompass.lshtm.ac.uk/1997/. All data generated or analysed during this study are included in this published article and its supplementary information files.
