# Supplementary material for: Evaluation of the InTray and Compact Dry culture systems for the diagnosis of urinary tract infections in patients presenting to primary health clinics in Harare, Zimbabwe
Source: Eur J Clin Microbiol Infect Dis. 2021 Jul 22;40(12):2543–50. doi: 10.1007/s10096-021-04312-4 (PMC8590652; doi:10.1007/s10096-021-04312-4)

**Journal:** European Journal of Clinical Microbiology & Infectious Diseases

**Title:** Evaluation of the InTray and Compact Dry culture systems for the diagnosis of urinary tract infections in patients presenting to primary health clinics in Harare, Zimbabwe

**Authors:** Ioana D Olaru^1,2^, Wael Elamin^3,4,5^, Mutsawashe Chisenga^2^, Nada Malou^6^, Jeremie Piton^6^, Shunmay Yeung^1,7^, Rashida A Ferrand^1,2^, Heidi Hopkins^1^, Prosper Chonzi^8^, Kudzai PE Masunda^8^, Portia Manangazira^9^, Cecilia Ferreyra^6^, Katharina Kranzer^1,2,10^

**Affiliations**

1. Clinical Research Department, London School of Hygiene and Tropical Medicine, London, United Kingdom
2. Biomedical Research and Training Institute, Harare, Zimbabwe
3. G42 Health Care, Abu Dhabi, United Arab Emirates
4. Queen Mary University London, London, United Kingdom
5. Elrazi University, Khartoum, Sudan
6. FIND (Foundation for Innovative New Diagnostics), Geneva, Switzerland
7. Department of Paediatric Infectious Disease, St Mary’s Imperial College Hospital, London, UK
8. City of Harare, Health Department, Harare, Zimbabwe
9. Ministry of Health and Child Care, Zimbabwe
10. Division of Infectious and Tropical Medicine, Medical Centre of the University of Munich, Munich, Germany

**Correspondence to:**

Ioana D Olaru; email: [ioana-diana.olaru@lshtm.ac.uk](mailto:ioana-diana.olaru@lshtm.ac.uk)

**Supplementary materials**

**Additional Table S1.** Characteristics of participants in a study of patients presenting with symptoms of urinary tract infection to primary care clinics in Harare, Zimbabwe

| **Characteristic** | **N=414** |
| --- | --- |
| Age, median (IQR) | 36 (26-46) |
| Female sex, n (%) | 263 (63.5) |
| Pregnant, n (%) | 32 (12.5) |
| HIV+, n (%) | 169 (42.7) |
| On co-trimoxazole prophylaxis, n (%) | 77 (45.6) |
| Urine dipstick positive, n (%) | 125 (30.3) |
| Leucocyturia on microscopy, n (%) | 81 (19.6) |
| Reported prior antimicrobials, n (%) | 14 (3.4) |

*This analysis excludes participants with contaminated urine cultures on Brilliance UTI agar (n=17). Missing information: women who did not know if they were pregnant (n=6); participants who did not know their HIV status (n=18); dipstick, urine microscopy and antibiotic assay could not be performed because of sample spillage during transport (n=1). Urine dipstick was considered positive if it was positive for nitrites and/or leucocytes.*

**Fig. S1** Appearance of different colony types on InTray Screen culture plates (from left to right 1. *E. coli;* 2. Coliforms; 3. *Enterococcus spp.*; 4. *Staphylococcus aureus*; 5. *Proteus mirabilis*; 6. Staphylococci/ streptococci (contaminants))


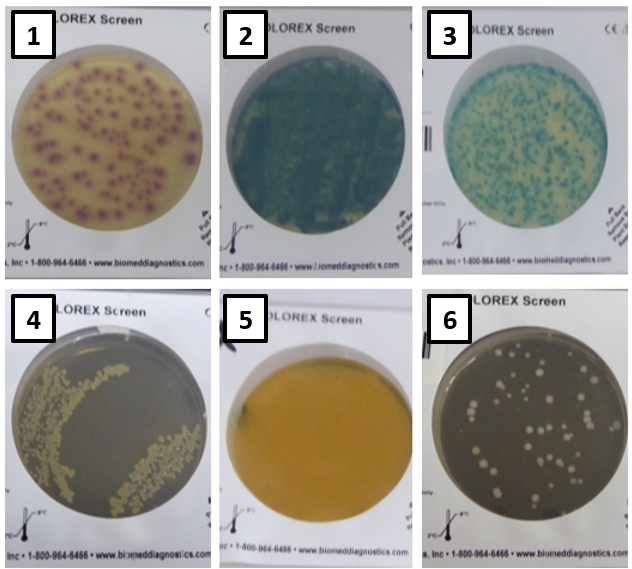


**Fig. S2** Appearance of E. coli and K. pneumoniae at different dilutions using Compact Dry EC


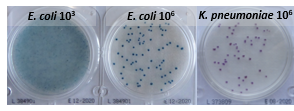

Supplement: Supplementary file 1 — Supplementary file1 (DOCX 452 KB) [file 10096_2021_4312_MOESM1_ESM.docx]
