# Supplementary material for: Evaluation of the InTray and Compact Dry culture systems for the diagnosis of urinary tract infections in patients presenting to primary health clinics in Harare, Zimbabwe
Source: Eur J Clin Microbiol Infect Dis. 2021 Jul 22;40(12):2543–50. doi: 10.1007/s10096-021-04312-4 (PMC8590652; doi:10.1007/s10096-021-04312-4)
Supplement: Supplementary file 2 — Supplementary file2 (DOCX 19 KB) [file 10096_2021_4312_MOESM2_ESM.docx]

**Journal:** European Journal of Clinical Microbiology & Infectious Diseases

**Title:** Evaluation of the InTray and Compact Dry culture systems for the diagnosis of urinary tract infections in patients presenting to primary health clinics in Harare, Zimbabwe

**Authors:** Ioana D Olaru^1,2^, Wael Elamin^3,4,5^, Mutsawashe Chisenga^2^, Nada Malou^6^, Jeremie Piton^6^, Shunmay Yeung^1,7^, Rashida A Ferrand^1,2^, Heidi Hopkins^1^, Prosper Chonzi^8^, Kudzai PE Masunda^8^, Portia Manangazira^9^, Cecilia Ferreyra^6^, Katharina Kranzer^1,2,10^

**Affiliations**

1. Clinical Research Department, London School of Hygiene and Tropical Medicine, London, United Kingdom
2. Biomedical Research and Training Institute, Harare, Zimbabwe
3. G42 Health Care, Abu Dhabi, United Arab Emirates
4. Queen Mary University London, London, United Kingdom
5. Elrazi University, Khartoum, Sudan
6. FIND (Foundation for Innovative New Diagnostics), Geneva, Switzerland
7. Department of Paediatric Infectious Disease, St Mary’s Imperial College Hospital, London, UK
8. City of Harare, Health Department, Harare, Zimbabwe
9. Ministry of Health and Child Care, Zimbabwe
10. Division of Infectious and Tropical Medicine, Medical Centre of the University of Munich, Munich, Germany

**Correspondence to:**

Ioana D Olaru; email: [ioana-diana.olaru@lshtm.ac.uk](mailto:ioana-diana.olaru@lshtm.ac.uk)

**Availability of data and materials**

The datasets generated and/or analysed during the current study are available in the Data Compass (LSHTM) repository which can be accessed from <https://datacompass.lshtm.ac.uk/1997/> .

**Data analysis codebook (for the deposited data)**

| **Variable name** | **Variable description** | **Variable type** | **Options** |
| --- | --- | --- | --- |
| Number | Identification for original dataset (annonymised) | integer | NOTE: number from 1 to 431 |
| Age_group | Age group (years) | categorical | Age bands of 5 years (excepting 18-24 years) |
| sex | Sex | categorical | 1: male  2: female |
| HIV status | Reported HIV status | categorical | 0: negative  1: positive  9: unknown/ not answered |
| Culture result | Culture result on the reference media | categorical | No growth  Contamination  10^3^-10^4^ CFU/ml  10^4^-10^5^ CFU/ml  10^5^ CFU/ml |
| Organism | Organism isolated from culture (positive cultures only: culture result =1) | categorical | *E. coli*  Coliforms  *Proteus, Providencia, Morganella* group  *Enterococcus spp.*  *S. aureus*  Missing : not applicable if culture negative/ contaminated |
| cefpodoxime | Antibiotic susceptibility test for cefpodoxime (for *Enterobacteriaceae* only = *E. coli*, Coliforms or *Proteus*, *Providencia* and *Morganella* group) | Categorical | 0: sensitive  1: resistant  Missing: not done if culture negative/ contaminated/ organism *Enterococcus spp* or *Staphylococcus spp.* |
| ESBL | Presence of extended-spectrum beta-lactamases  IF organism is *Enterobacteriaceae* (*E. coli*, Coliforms or *Proteus*, *Providencia* and *Morganella* group)  AND cefpodoxime =1 (resistant) | Categorical | Absent  Present  Missing: not done if organism is not *Enterobacteriaceae* and cefpodoxime is sensitive |
| Intray taken out | Number of times the InTray was taken out of the refrigerator to be sent to the field | Integer | -  Missing: missing value |
| Intray result | Culture result using the InTray screen | categorical | 0: no growth  1: positive culture with a pathogen  2: some growth of non-pathogenic bacteria or insignificant growth of a pathogen (<5 CFU)  3: growth of a non-pathogenic bacteria (more than at #2) |
| Intray organism | Organism identified using the InTray (only for positive cultures with pathogenic bacteria: InTray result = “1”) | categorical | *E. coli*  Coliforms  Proteus (includes *Proteus, Providencia, Morganella*)  *Enterococcus spp.*  *S. aureus*  Missing: culture negative on InTray/ contamination/ growth of non-pathogenic organisms |
| Intray growth (enterobacteria) | Growth on InTray (classification applicable to enterobacteria) | categorical | 0: negative/ insignificant growth  1: 5-49 CFU/ plate  2: 50-100 CFU/ plate  3: confluent growth (CFUs cannot be counted)  Missing: culture is contaminated on Brilliance UTI agar |
| CD 103 | Number of colonies on Compact Dry at 1: 10^3^ dilution | integer | Note:  999: semi-confluent growth  9999: confluent growth |
| CD 106 | Number of colonies on Compact Dry at 1: 10^6^ dilution | integer | Note:  999: semi-confluent growth  9999: confluent growth |
| CD category 103 | Growth on Compact Dry at dilution 10^3^  (this is a variable derived from CD 103) | categorical | 0: negative/ insignificant growth  5: 5-49 CFU/ml  50: 50-250 CFU/ml  250: >250 colonies/ semi-confluent growth  9998 : confluent growth |
| CD category 106 | Growth on Compact Dry at dilution 10^6^  (this is a variable derived from CD 106) | categorical | 0: negative/ insignificant growth  5: 5-49 CFU/ml  50: 50-250 CFU/ml  250: >250 colonies/ semi-confluent growth  9998: confluent growth |
| CD positive | Growth on Compact Dry | categorical | 0: no growth  1: growth (significant) |

*CFU: colony forming units*
